# Chromosome-level genome assembly of *Sinodiscula camellicola* strain Scam5

**DOI:** 10.1038/s41597-026-07115-4

**Published:** 2026-04-20

**Authors:** Xingyun Shi, Zehong Meng, Shuai Li, Xiuxian Shen, Jianfeng Jin, Zhong Li, Yufeng Zhou, Jinfeng Zhang

**Affiliations:** 1 Key Laboratory of Agricultural Microbiology, College of Agriculture, Guizhou University, Guiyang, 550025 China; 2 Green Control and Quality Safety Functional Laboratory of Guizhou Modern Agricultural Industry Technology System of Tea, Guizhou Tea Research Institute, Guiyang, 550006 China; 3 Institute of Entomology, Guizhou Key Laboratory of Agricultural Biosecurity, College of Agriculture, Guizhou University, Guiyang, 550025 China; 4 College of Life Sciences, Xinyang Normal University, Xinyang, 464000 China; 5 Guizhou Key Laboratory of Agricultural microbiology, Guizhou Academy of Agricultural Sciences, Guiyang, 550006 China

**Keywords:** Eukaryote, Fungal genomics

## Abstract

*Sinodiscula camellicola* is the causative agent of tea anthracnose, leading to severe leaf withering and defoliation. This disease compromises plant growth, reduces yield and quality, and causes substantial economic losses to the tea industry. However, research into the pathogenic mechanisms, host adaptation, and evolution of *S. camellicola* has been hampered by the scarcity of high-quality genome assemblies. By integrating Illumina short-read, PacBio HiFi, and Hi-C sequencing data, we generated a chromosome-level genome assembly for *S. camellicola*. The 41.79 Mb genome is organized into nine scaffolds and nine contigs (41.79 Mb, 100%), all anchored to nine chromosomes, with a scaffold N50 of 4.45 Mb. Using the fungi_odb10 dataset (n = 758), BUSCO analysis revealed a genome assembly completeness of 98.8%, including 98.7% single-copy genes and 0.1% duplicated genes. A total of 2,978,299 bp of repeat sequences and 9,918 protein-coding genes were predicted. The high-quality genome assembly of *S. camellicola* provides critical insights into its pathogenic mechanisms and supports the development of science-based strategies for effective disease management in agriculture.

## Background & Summary

Tea [*Camellia sinensis* (L.) O. Kuntze] is a perennial evergreen leaf-producing plant whose young shoots are commonly processed into various widely consumed tea products^1,2^. The tea plant contains a wide range of beneficial compounds, including polyphenols, theanine, and other bioactive ingredients that contribute to human health. Owing to its broad applications and significant economic value, tea is commercially cultivated in more than 60 countries worldwide^3,4^. According to FAO statistics, approximately 4.7 million hectares were planted with tea globally in 2023 (https://www.fao.org/faostat/). China accounts for the largest share, with 2.95 million hectares across 20 provinces, representing 61% of the global total^5^. However, tea cultivation and production are threatened by numerous harmful factors, including diseases, pests, and weeds. These biotic stresses severely affect yield and quality, resulting in substantial economic losses. Globally, nearly 380 types of tea diseases have been documented. In China, approximately 100 species have been identified, more than 40 of which are considered common, with the majority being leaf diseases^6,7^. Among these, tea anthracnose is a major leaf disease that causes extensive necrosis and leaf abscission. This disease adversely affects plant growth, leading to reduced yield and lower quality. It is estimated that tea anthracnose causes approximately 20% production loss in tea cultivation^8,9^.

The etiology of tea anthracnose has historically been contentious. Although the *Colletotrichum gloeosporioides* species complex (particularly *C. camelliae* and *C. fructicola*) has frequently been associated with tea leaf diseases, recent rigorous taxonomic and pathological re-evaluations have clarified a critical distinction^10–12^. *Colletotrichum* species are primarily responsible for tea brown blight, a disease characterized by distinct symptomatology and infection biology^13,14^. The true causal agent of classical tea anthracnose—characterized by specific necrotic lesions first described by Miyake—has been identified as the fungus historically known as *Gloeosporium theae-sinensis* (later *Discula theae-sinensis*)^15–17^. In 2024, this pathogen was reclassified into the new genus *Sinodiscula* (family Melanconiellaceae, order Diaporthales), which includes species such as *S. theae-sinensis* and the newly described *S. camellicola*^18–20^. Unlike *Colletotrichum*, which utilizes melanized appressoria for direct cuticular penetration, *Sinodiscula* species uniquely employ a trichome-mediated entry pathway to infect host leaves^21,22^.

Despite the confirmed pathogenicity and distinct evolutionary lineage of *S. camellicola*, genomic resources for this pathogen have been lacking. Previous genomic studies in tea pathology have focused almost exclusively on *Colletotrichum* species^23,24^. This gap has limited our understanding of the molecular mechanisms underlying non-appressorial infection and has impeded the development of targeted control strategies.

Here, we present the first high-quality, chromosome-level genome assembly of *Sinodiscula camellicola* strain Scam5, generated using an integrated approach combining Illumina short-read, PacBio HiFi long-read, and Hi-C sequencing technologies. The genome was assembled into nine pseudochromosomes totaling 41.79 Mb, with a GC content of 51.09%, and 9,918 protein-coding genes were annotated.

To highlight the biological distinctiveness and potential reuse value of this genomic resource, we provide a comparative genomic context with representative *Colletotrichum* tea pathogens (Table 1). The *S. camellicola* genome is substantially more compact (41.79 Mb) than those of *C. camelliae* (67.74 Mb) and *C. fructicola* (56.8 Mb) and contains approximately 33% fewer predicted genes. This apparent genomic streamlining may reflect its specialized hemibiotrophic lifestyle and adaptation to the trichome niche, in contrast to the expanded gene families observed in broader host-range *Colletotrichum* species. Furthermore, whereas available *Colletotrichum* genomes are often fragmented (e.g., 464 scaffolds for *C. fructicola*), our chromosome-level assembly enables precise mapping of structural variations and gene clusters that are frequently obscured in fragmented assemblies.

**Table 1:** Comparison of genome features between *Sinodiscula camellicola* and representative tea-associated *Colletotrichum* species.

Species	Disease on tea	Assembly level	Genome size (Mb)	No. of chromosomes or contigs/scaffolds	GC content (%)	Protein-coding genes	Reference
*S. camellicola*	Anthracnose	Chromosome-level (Hi-C)	41.79	9	51.09	9918	This study
*C. camelliae*	Tea brown blight	Contig-level	67.74	21	48.98	14849	^23^
*C. fructicola*	Tea leaf spot	Scaffold-level	56.8	464	53.45	15243	^24^

## Methods

### Sample collection and sequencing

**Fig. 1:**
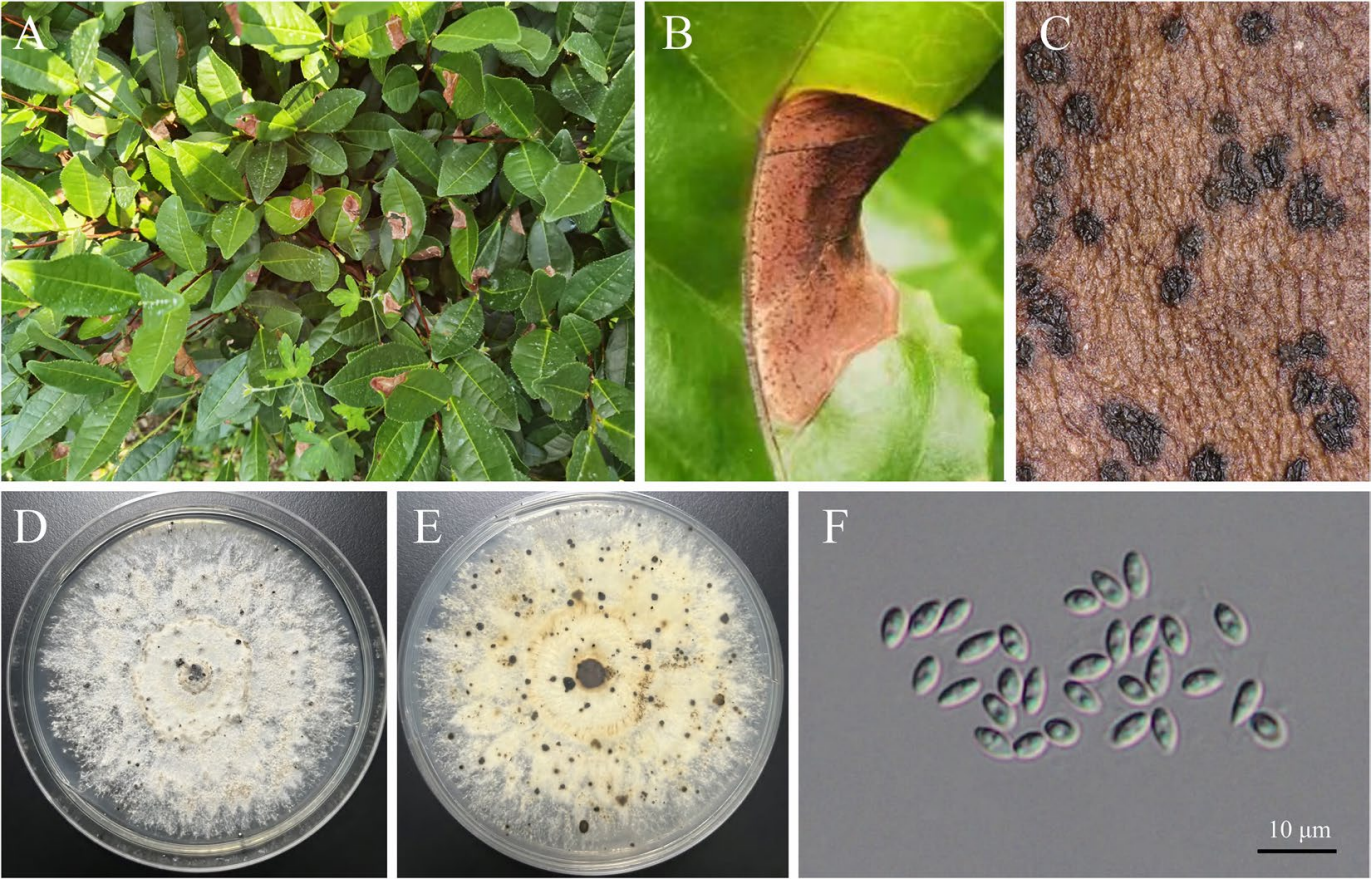
Tea (*Camellia sinensis*) anthracnose disease and morphology of the fungal strain Scam5. (**A**) *S. camellicola* causes a large number of fallen leaves of tea. (**B**) Lesion on the tea. (**C**) Habit of conidiomata on the leaf of tea. (**D,E**) Upper and reverse sides of cultures. (**F**) Conidia. Scale bars are 10 μm.

The *S. camellicola* strain Scam5 used in this study was isolated on May 20, 2021, from diseased tea leaves collected in Wende Village, Duyun City, Qiannan Buyi and Miao Autonomous Prefecture, Guizhou Province, China (26°24′17″N, 107°29′42″E) (Fig. 1A–C). The morphological characteristics of the isolate were consistent with those previously described for *S. camellicola*^18^ (Fig. 1D–F). The Scam5 strain was cultured on potato dextrose agar medium under a 12 h light/12 h dark photoperiod at 25 °C for 15 days. Mycelia were then harvested, flash-frozen in liquid nitrogen for 10 min, and stored at −80 °C until DNA and RNA extraction.

High-molecular-weight genomic DNA was extracted using the hexadecyl trimethyl ammonium bromide method. Total RNA was extracted using TRIzol™ Reagent (Thermo Fisher Scientific) according to the manufacturer’s instructions. DNA and RNA were obtained from cultures grown under identical conditions. For Illumina sequencing, libraries were prepared using the TruSeq DNA PCR-Free Kit, generating paired-end reads of 150 bp with an insert size of 350 bp. High-throughput chromosome conformation capture (Hi-C) libraries were constructed following a previously published protocol^25^, which included DNA cross-linking, chromatin digestion with *MboI*, end repair, DNA ligation, and purification. Short-read sequencing was performed on the Illumina NovaSeq. 6000 platform. For long-read sequencing, a 15 kb insert library was constructed using the SMRTbell™ Express Template Prep Kit 2.0 and sequenced on the PacBio Sequel IIE platform in HiFi mode. All library preparation and sequencing were conducted by Berry Genomics (Beijing, China). In total, 20.46 Gb of sequencing data were generated, including 3.77 Gb (90.22 × coverage) from PacBio HiFi reads, 5.34 Gb (127.75 × coverage) from Illumina reads, and 6.24 Gb (149.30 × coverage) from Hi-C data (Table 2).

**Table 2:** Statistics of the sequencing data used for genome assembly.

Libraries	Insert sizes (Kb)	Clean data (Gb)	Sequencing coverage (×)
Illumina	0.35	5.34	127.75
PacBio HiFi	15	3.77	90.22
Hi-C	0.35	6.24	149.30
RNA	0.35	5.11	—

### Genome assembly

**Fig. 2:**
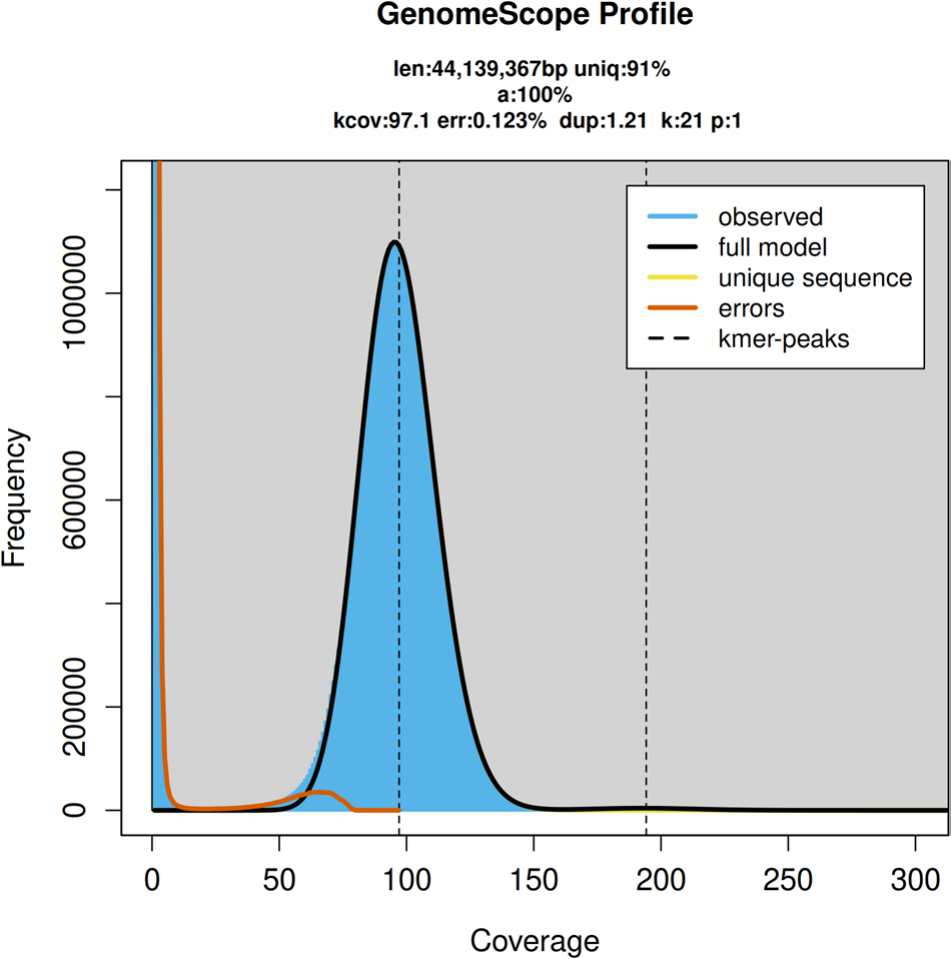
Genome size estimation for *Sinodiscula camellicola* based on GenomeScope.

To ensure quality control of the raw Illumina data, we used BBTools v38.82^26^. Duplicate reads were removed using the “clumpify.sh” script. Read quality was further improved using the “bbduk.sh” script, which discarded sequences with quality scores below 20, filtered out reads containing more than five ambiguous bases (Ns), trimmed poly-A/G/C tails longer than 10 bp, and corrected overlaps in paired-end reads. To estimate genome size, heterozygosity, and repetitive sequence content in *S. camellicola*, we performed a genome survey using GenomeScope v2.0^27^. K-mer frequency analysis was conducted with khist.sh (BBTools) using a k-mer size of 21. Based on k-mer coverage and frequency distribution, the genome size of *S. camellicola* was estimated to be approximately 44.14 Mb (Fig. 2).

**Fig. 3:**
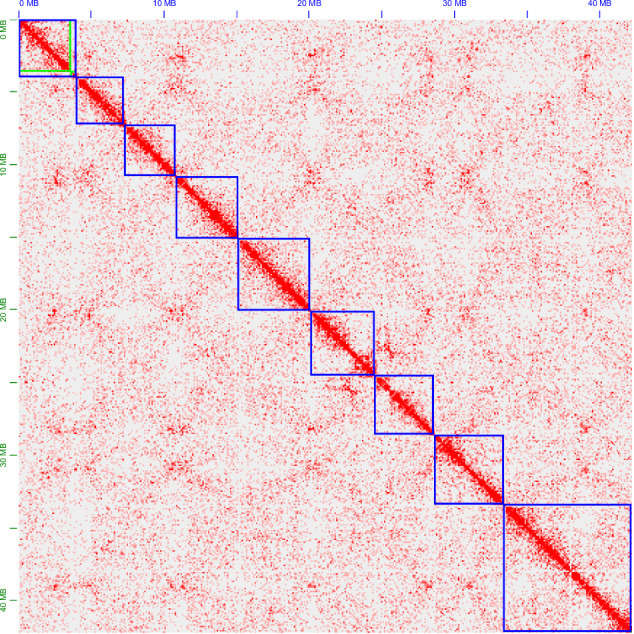
The chromosomal heatmap visualization of *Sinodiscula camellicola* genome assembly displays complete chromosomes in blue, with individual contigs demarcated by green borders.

**Fig. 4:**
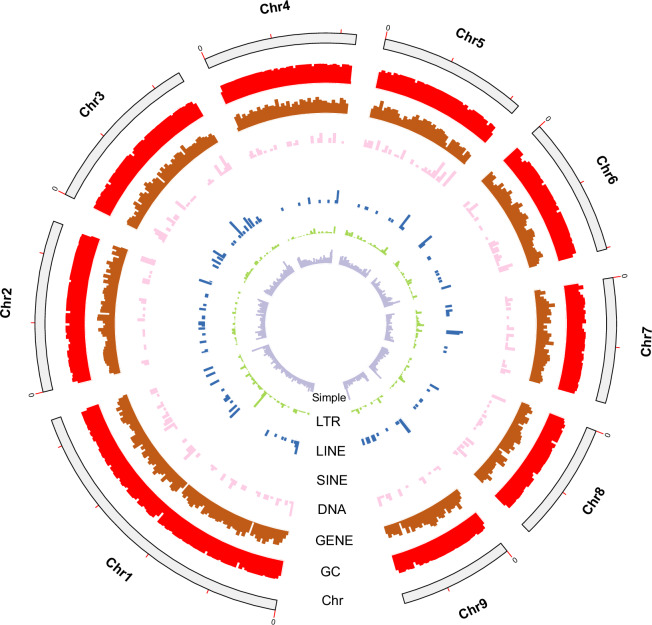
The genomic features of *Sinodiscula camellicola* are displayed in a circular layout. Moving inward from the outermost ring, the visualization depicts (1) chromosome length, (2) GC content, (3) gene density, and (4) various repetitive elements, including transposable elements (DNA, SINEs, LINEs, and LTRs), along with simple repeat sequences.

High-quality HiFi reads were initially assembled using Hifiasm v0.16.1^28^ with the parameter “-l 2,” corresponding to the aggressive mode for removing redundant (heterozygous) sequences. To minimize potential contamination or assembly artifacts, only contigs with sequencing depth greater than 10 × were retained. Prior to Hi-C–based scaffolding, Hi-C sequencing data were quality-controlled using Juicer v1.6.2^29^. Chromosome anchoring and scaffolding were subsequently performed using 3D-DNA v180922^30^. To validate assembly accuracy, contig alignment results were carefully examined, and potential misassemblies were manually corrected using Juicebox v1.11.08^29^. To identify potential contaminants in the assembled genome, MMseqs. 2 v13^31^ was used to perform sequence similarity searches comparable to BLASTN against the National Center for Biotechnology Information (NCBI) nucleotide and UniVec databases. In addition, BLAST + v2.11.0^32^ was employed to screen for vector contamination by comparison with the UniVec database. Sequences exhibiting more than 90% similarity to entries in these databases were considered potential contaminants. Sequences with greater than 80% similarity were further verified through BLASTN searches against the NCBI nucleotide database. No significant contamination was detected, indicating high sample quality and sequencing accuracy. The final chromosome-scale assembly of *S. camellicola* spans 41.79 Mb and comprises nine scaffolds and nine contigs, consistent with the genome size estimated from the genome survey. The assembly demonstrated remarkable continuity, with scaffold and contig N50 values of 4.45 Mb (Table 3; Table 4). All assembled sequences (41.79 Mb) were successfully anchored to nine chromosomes (Fig. 3; Fig. 4). The inferred chromosome number (nine) is consistent with that of *Sinodiscula theae-sinensis*, a congeneric species whose genome is available under NCBI accession GCA_051027275.1. BUSCO analysis further indicated a genome assembly completeness of 98.8% (Table 3). Collectively, these results demonstrate that the assembled genome exhibits high continuity, completeness, and structural integrity.

**Table 3:** Genome assembly statistics for *Sinodiscula camellicola*.

Assembly	Total length (Mb)	Number scaffolds/contigs (chromosomes)	Scaffold/contig N50 length (Mb)	GC (%)	BUSCO (fungi_odb10, n = 758) (%)			
					C	D	F	M
Hifiasm	43.39	15/15	4.45/4.45	51.09	98.8	0.1	0.1	1.1
3D-DNA	43.39	13/15 (9)	4.45/4.45	51.09	98.8	0.1	0.1	1.1
Final	41.79	9/9 (9)	4.45/4.45	51.41	98.8	0.1	0.1	1.1

C: complete BUSCOs; D: complete and duplicated BUSCOs; F: fragmented BUSCOs; M: missing BUSCOs.

**Table 4:** Genome assembly and annotation statistics of *Sinodiscula camellicola*.

Characteristics	*S. camellicola*
Genome assembly	
Genome Size (Mb)	41.79
Number of scaffolds	9
Number of chromosomes	9
Scaffold N50 length (Mb)	4.45
GC (%)	51.09
BUSCO completeness (%)	98.8
Protein-coding genes	
Number	9,918
Mean gene length (bp)	2333.7
BUSCO completeness (%)	99.6
Repetitive elements	
Size (Mb)	2.98 (7.13%)
DNA transposons (Mb)	0.06 (0.12%)
LINEs (Mb)	0.02 (0.04%)
LTRs (Mb)	1.36 (3.26%)
Unclassified (Mb)	0.78 (1.87%)
Simple repeat (Mb)	0.48 (1.14%)
ncRNA	
Number of ncRNA	230
rRNA	95
tRNA	91
snRNA	25

### Genome annotation

To characterize repetitive elements in the *S. camellicola* genome, de novo repeat annotation was performed using RepeatModeler v2.0.4^33^. The resulting repeat library was combined with RepBase (release 20230909)^34^ and Dfam v3.5^35^ to construct a custom repeat database, which was subsequently used by RepeatMasker v4.1.2^36^ to identify and mask repetitive sequences. Repetitive elements accounted for 7.13% of the genome assembly. These were classified into major categories, including 3.26% long terminal repeat (LTR) elements, 1.87% unclassified repeats, 1.14% simple repeats, and other repeat types (Table 4).

Non-coding RNAs (ncRNAs) were identified using Infernal v1.1.4^37^ with the Rfam v14.10^38^ database, and tRNAs were predicted using tRNAscan-SE v2.0.9^39^. The findings indicated a rich variety of ncRNAs. A total of 230 ncRNAs were identified, including 91 transfer RNAs (tRNAs), 95 ribosomal RNAs (rRNAs), and 25 small nuclear RNAs (snRNAs) (Table 4).

Protein-coding gene annotation in the *S. camellicola* genome was performed using MAKER v3.01.03^40^, integrating transcriptomic evidence, ab initio predictions, and protein homology information. Transcriptome reads were aligned to the genome using HISAT2 v2.2.0^41^, followed by genome-guided transcript assembly with StringTie v2.1.6^42^. Ab initio gene prediction was conducted using BRAKER v2.1.6^43^, which incorporates GeneMark-ES/ET/EP 4.68-3.60_lic^44^ and Augustus v3.3.4^45^. Homology-based gene prediction was performed using GeMoMa v1.8^46^. Reference protein sequences were obtained from closely related Ascomycota species, including *Podospora pseudopauciseta* (GCF_035222475.1), *Pyricularia pennisetigena* (GCA_004337985.1), *Remersonia thermophila* (GCA_042764415.1), and *Sordaria macrospora* (GCA_000182805.2). In total, 9,918 protein-coding genes were predicted in the *S. camellicola* genome, with an average gene length of 2,333.7 bp (Table 4). On average, each gene contained 3.2 exons, 2.1 introns, and 3.1 coding sequences (CDSs). The mean lengths of exons, introns, and CDSs were 663.4 bp, 115.6 bp, and 513.0 bp, respectively. To assess the quality of gene predictions, gene set completeness was evaluated using BUSCO with the fungal dataset (fungi_odb10, n = 758). The analysis yielded a completeness score of 99.6% (Table 4), indicating a highly complete protein-coding gene set.

Functional annotation was performed by aligning predicted protein sequences to the UniProtKB (Swiss-Prot and TrEMBL) database using DIAMOND v2.0.11.149^47^. Gene Ontology (GO) terms, KEGG and Reactome pathways, and protein domains were annotated using eggNOG-mapper v2.1.5^48^ and InterProScan 5.53-87.0^49^. InterProScan integrated data from four databases: Pfam^50^, SMART^51^, Superfamily^52^, and CDD^53^. Based on combined InterProScan and eggNOG annotations, 8,251 COG categories, 6,341 GO terms, 2,100 enzyme codes, and 2,753 KEGG pathways were identified in *S. camellicola* (Table 5). Chromosomal features, including repetitive element distribution, gene density, and GC content, were visualized using TBtools v1.098769^54^.

**Table 5:** Genome function annotation statistics of *Sinodiscula camellicola*.

Function annotation	Number
Number of genes matching Uniprot records	9,553
Number of genes with InterProScan annotations	8,264
Number of genes with GO items from InterProScan annotations	5,142
Number of genes from eggNOG annotations	
gene names (function)	9,441
GO items	3,819
Enzyme Codes (EC)	2,100
KEGG ko terms	4,351
KEGG pathway terms	2,753
COG Functional Categories	8,251
Number of genes with GO items (combining InterProScan and eggNOG results)	6,341
Number of genes with KEGG pathways items items (combining InterProScan and eggNOG results)	2,753

## Data Records

The raw sequencing data generated in this study have been deposited in the NCBI under BioProject accession number PRJNA1280708. The Hi-C, transcriptome, Illumina, and PacBio HiFi sequencing datasets are available through the Sequence Read Archive under accession numbers SRR35179660–SRR35179663^55–58^. The final genome assembly has been submitted to GenBank and is accessible under accession number GCA_053477585.1^59^. Annotation files, including repeat sequences, gene structures, and functional predictions, have been deposited in Figshare^60^.

## Technical Validation

To evaluate the quality of the assembled genome, two complementary approaches were employed. First, assembly completeness was assessed using BUSCO v5.0.4^61^ with the fungal reference dataset (fungi_odb10, n = 758). The genome assembly achieved a BUSCO completeness score of 98.8%, comprising 98.7% single-copy genes, 0.1% duplicated genes, 0.1% fragmented genes, and 1.1% missing genes (Table 3). Second, read alignment rates were calculated to further assess assembly accuracy. The mapping rates of PacBio HiFi, Illumina, and RNA-seq reads to the assembled genome were 91.71%, 92.62%, and 94.74%, respectively.

## Data Availability

The raw sequencing reads and the assembled genome of *Sinodiscula camellicola* have been deposited in the NCBI database under BioProject accession number PRJNA1280708. In addition, annotation files, including repeat elements, gene structures, and functional predictions, are available via Figshare (10.6084/m9.figshare.30022063).

## Code availability

No custom code was used in this study. All analyses were performed using publicly available software packages, as described in the Methods.
